# Metazoan parasite communities of two deep-sea elasmobranchs: the southern lanternshark, *Etmopterus granulosus*, and the largenose catshark, *Apristurus nasutus*, in the Southeastern Pacific Ocean

**DOI:** 10.1051/parasite/2018054

**Published:** 2018-11-20

**Authors:** Juan F. Espínola-Novelo, Rubén Escribano, Marcelo E. Oliva

**Affiliations:** 1 Programa de Doctorado en Ciencias Aplicadas, mención Sistemas Marinos Costeros, Universidad de Antofagasta P.O. Box 179 Antofagasta Chile; 2 Departamento Oceanografía, Universidad de Concepción P.O. Box 160-C Concepción Chile; 3 Millennium Institute of Oceanography (IMO), Universidad de Concepción P.O. Box 160-C Concepción Chile; 4 Instituto de Ciencias Naturales Alexander von Humboldt, Universidad de Antofagasta P.O. Box 170 Antofagasta Chile

**Keywords:** Deep-sea sharks, metazoan parasites, biodiversity, Southeastern Pacific Ocean

## Abstract

Two deep-sea shark species were obtained as by-catch of the local fishery of the Patagonian toothfish, *Dissostichus eleginoides*, at depths ranging from 1000 to 2200 m off central and northern Chile. A total of 19 parasite taxa were found in 133 specimens of the southern lanternshark, *Etmopterus granulosus*, (*n* = 120) and largenose catshark, *Apristurus nasutus,* (*n* = 13). Fourteen taxa (four Monogenea, one Digenea, four Cestoda, one Nematoda, two Copepoda, one Annelida and one Thecostraca) were found in *E. granulosus*, whereas five taxa (one Monogenea, two Cestoda and two Nematoda) were found in *A. nasutus*. Representatives of Cestoda showed higher values of prevalence and a greater intensity of infection; this pattern is consistent with reports for elasmobranchs, but the monogenean richness was higher than that previously reported for related deep-sea sharks. Regarding *E. granulosus*, a positive and significant correlation between host length and abundance was found for six (four ectoparasites, one mesoparasite, and one endoparasite) of the 14 taxa recorded, but prevalence was significantly correlated with host length only for the monogenean *Asthenocotyle* sp. Although the sample size for *A. nasutus* was limited, we compared richness, abundance, diversity and evenness at the infracommunity and component community levels. All of these variables were higher for *E. granulosus*, but at the infracommunity level, abundance was higher for *A. nasutus*. All the parasite taxa (except two) represent new host and geographical records.

## Abstract (fr)

Deux espèces de requins des grands fonds ont été obtenues comme prises accessoires de la pêcherie locale de Légine australe, *Dissostichus eleginoides*, à des profondeurs allant de 1000 à 2200 m, au large du centre et du nord du Chili. Au total, 19 taxons de parasites ont été trouvés dans 133 spécimens du Sagre long nez *Etmopterus granulosus* (*n* = 120) et du Holbiche cyrano *Apristurus nasutus* (*n* = 13). Quatorze taxons (quatre Monogenea, un Digenea, quatre Cestoda, un Nematoda, deux Copepoda, un Annelida et un Thecostraca) ont été trouvés chez *E. granulosus*, tandis que cinq taxons (un Monogenea, deux Cestoda et deux Nematoda) ont été trouvés chez *A. nasutus*. Les représentants des Cestoda ont montré des valeurs de prévalence plus élevées et une plus grande intensité d’infection; ce schéma concorde bien avec ce qui a été rapporté pour les élasmobranches, mais la richesse des Monogenea était supérieure à celle précédemment rapportée pour les requins de profondeur apparentés. En ce qui concerne *E. granulosus*, une corrélation positive et significative entre la longueur de l’hôte et l’abondance a été constatée pour six des 14 taxons répertoriés (quatre ectoparasites, un mésoparasite et un endoparasite), mais la prévalence était significativement corrélée à la longueur de l’hôte uniquement pour le Monogène *Asthenocotyle* sp. Bien que la taille de l’échantillon d’*A. nasutus* soit limitée, nous avons comparé la richesse, l’abondance, la diversité et la régularité aux niveaux infracommunautaire et communautaire. Toutes ces variables étaient plus élevées pour *E. granulosus*, mais au niveau infracommunautaire, l’abondance était supérieure pour *A. nasutus*. Tous les taxons parasites, à l’exception de deux, représentent de nouvelles mentions géographiques et de nouveaux hôtes.

## Introduction

The deep-sea (>200 m depth) is the habitat for 10–15% of the global ichthyofauna [29, 30]. Despite its limited primary production, important marine resources inhabit this environment [3]; consequently, as coastal fisheries have collapsed, the deep-sea region may offer new resources [41]. Accordingly, we need not only clear and adequate knowledge of these potential new resources but also a sound understanding of the biodiversity of these ecosystems [3, 29]. Parasites are an important component of any ecosystem, not only because of the number of species (which is higher than the number of free-living species) but also because of the role they play in the trophic web [16, 31]. Deep-sea fish parasites have often been neglected, although they are an essential part of marine biodiversity [29]. Moreover, through the study of parasites, it is possible to obtain information about their hosts, such as their diet, migratory movements and population structures, and the biodiversity and human impacts in the ecosystems where they coexist [34, 56, 59]. Consequently, parasites can provide crucial clues about the features of the ecosystems inhabited by their hosts.

Knowledge of the parasite fauna harboured by deep-sea fishes is available for less than 10% of the ichthyofauna that inhabit these ecosystems [29, 30]. This is due to the economic and logistical difficulties in accessing the hosts that inhabit these ecosystems in comparison with fishes of commercial importance or those that inhabit coastal areas [30]; an alternative is to study hosts from the by-catch of deep-sea commercial species. Knowledge regarding the parasites of sharks is scarce [9], and it is even scarcer for the species that inhabit the deep-sea. Of the 509 species of sharks known to date, no fewer than 250 are considered deep-sea species [58]. Parasitological studies of species that inhabit these ecosystems focus on the taxonomic description of some species or new host records, but few include the analysis of the parasite community [14, 15, 25, 28]. For the Southeastern Pacific Ocean (SEPO herein and after), the same scenario is evident. Of the 68 shark species that have been reported, at least 33 are considered deep-sea sharks [17]. Only five studies have been carried out on the parasites of deep-sea sharks for the SEPO [11, 23, 32, 53, 54]. These papers focus on the taxonomic description of some parasite species or the presence of a parasite in a host; so far, no study has been conducted to address the metazoan parasite communities of deep-sea sharks for the SEPO.

*Etmopterus granulosus* (Günther, 1880), the southern lanternshark, and *Apristurus nasutus* De Buen, 1959, the longnose catshark, are two species inhabiting the deep-sea in the SEPO; *E. granulosus* shows a wide distribution in the Southern Ocean, including the southern Indian, southern Pacific and southwestern Atlantic Oceans, whereas *A. nasutus* is found from the Gulf of Panama to central Chile [17]. Both species are caught as by-catch in the local fishery of the Patagonian toothfish, *Dissostichus eleginoides* Smitt, 1898, in central and northern Chile as well as by-catch of the orange roughy, *Hoplostethus atlanticus* Collett 1889, in the Juan Fernandez Archipelago. Our goal is to report, for the first time, the composition of the metazoan parasite communities of two deep-sea sharks from the SEPO, as well as to quantitatively describe the characteristics of their parasite community.

## Materials and methods

**Figure 1.**
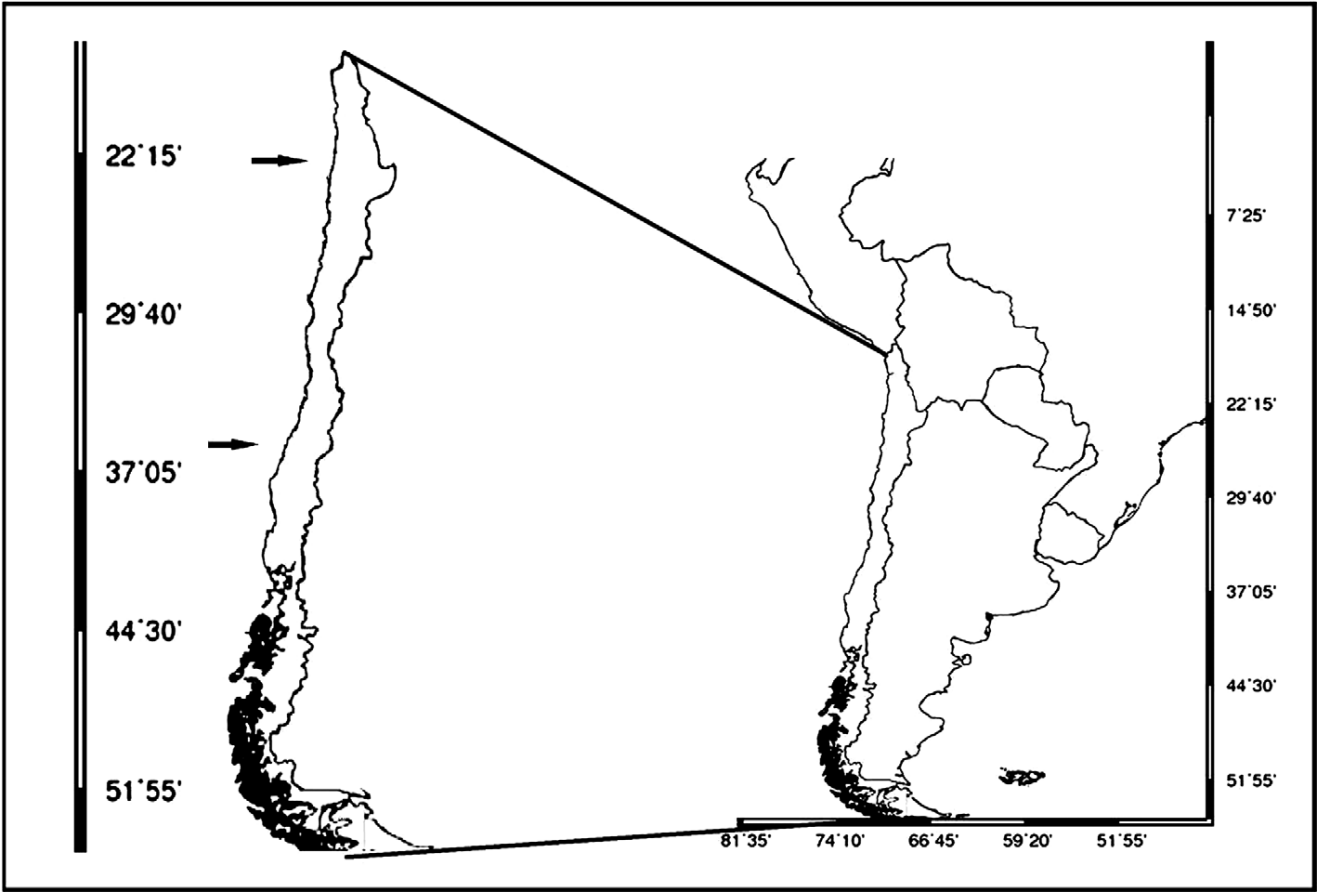
Approximate position of localities where samples of deep sea sharks were caught. Arrows indicate the approximate position of localities where samples of deep sea sharks were caught.

A total of 133 specimens (*E. granulosus* = 120, *A. nasutus* = 13) of deep-sea sharks were obtained during 2015–2017 (see Supplementary material) from the by-catch of the local fishery of the Patagonian toothfish (*D. eleginoides*) along the northern (22°16′S 70°38′W–23°26′S 70°43′W) and central (35°5′S–72°53′W) Chilean coasts at depths ranging from 1000 to 2200 m (Fig. 1) using a deep-sea longline. The sharks were captured, stored in bags and immediately frozen (−18 °C) on board and transported to the laboratory for parasitological analyses.

After thawing, the sharks were measured (total length to the nearest 1.0 cm), dissected and examined for metazoan parasites (both ectoparasites and endoparasites). Parasites were recorded by species and abundance for each shark, fixed in AFA (alcohol: formalin: acetic acid), and then preserved in 70% alcohol. Nematoda were cleared with Amann lactophenol. Digenea, Monogenea and Cestoda were stained (acetic carmine) and cleared with clove oil (Sigma-Aldrich) and then mounted in Eukitt medium (O. Kindler GmbH, Germany) [42]. Copepoda and Thecostraca were stored in ethanol (70%) and dissected for taxonomic purposes. Parasites were identified to the lowest taxonomic level possible. The prevalence and mean intensity of infection were calculated [8].

The quantitative analysis was carried out at the infracommunity and community component levels. For the infracommunity level, we calculated the richness (number of parasite species per examined host), abundance (number of parasite individuals per examined host) and diversity (Brillouin index) using PRIMER v6, parasite species accumulation curves were constructed using the “vegan” package in R freeware [44]. The Berger-Parker dominance index was calculated as the number of individuals of the most abundant parasite species divided by the total number of parasites in a given fish host as indicated by Dallarés et al. [14]. The descriptors Shannon, Simpson and Inverse Simpson diversity index were calculated at the community component level using the “vegan” package in R freeware. Because the sample size for *A. nasutus* was small, the characteristics were described but not compared, as 38% of the specimens of *A. nasutus* were not parasitized.

Potential relationships between host body length and richness (number of species), abundance (total number of individuals) and prevalence (previous angular transformation of prevalence data) were explored using the Spearman correlation coefficient only for *E. granulosus*. The potential relationship between length and abundance was explored in the same manner. Because of the small sample size of *A. nasutus*, the quantitative analysis of its parasite fauna was not performed. The statistical analyses were performed with the Minitab 17 statistical software program and Primer v6 [1].

## Results

A total of 120 specimens of *E. granulosus* and 13 specimens of *A. nasutus* were obtained from nine fishing events, two from the central and seven from the northern fishing zone. Because the sample size was small for some periods, seasonality was not analysed (Supplementary material). The sharks were obtained with the same fishing gear.

The total length for *E. granulosus* ranged from 24 to 96 cm (*M* = 46.1 ± 8.6), whereas the length range for *A. nasutus* was 52.5–84 cm (*M* = 65.1 ± 8.4). ANOVA showed that the mean length of the hosts differed between localities, but marginally (*F*_1, 118_ = 3.98, *p* = 0.048). Because the correlations between host length and total richness and total abundance were significant for both localities, the samples from northern and central sites were pooled.

A total of 277 parasite specimens belonging to 19 parasite taxa were obtained for the two hosts. Higher richness was found in *E. granulosus*, with 14 taxa: six endoparasites (one Digenea, four Cestoda and one Nematoda), seven ectoparasites (four Monogenea, two Copepoda, and one Annelida) and one mesoparasite (Thecostraca). Five taxa were found from *A. nasutus*; four were endoparasites (two Cestoda and two Nematoda) and one ectoparasite (Monogenea).

Four taxa of Monogenea were found in *E. granulosus* but with low values of prevalence and mean intensity. The monogenean *Microbothrium* sp. was found in *A. nasutus*, showing the highest mean intensity observed in this study (Table 1).

**Table 1.** Prevalence (P) and mean intensity ± standard deviation (*MI* ± *SD*) of infection of metazoan parasites found in two species of deep-sea sharks from the SEPO.

			*E. granulosus*		*A. nasutus*	
Parasite species	Stage	Habitat	P	*MI* ± *SD*	P	*MI* ± *SD*
Monogenea						
*Squalonchocotyle aff. spinacis*	A	G	5.1	1.2 ± 0.4	–	–
*Asthenocotyle* sp.	A	S	9.3	1.5 ± 0.5	–	–
*Microbothrium* sp.	A	S	–	–	15.3	24.0 ± 3.0
*Calicotyle* sp.	A	S	1	2 ± 0	–	–
*Monocotylidae* gen. sp.	A	S	3.4	1.5 ± 0.6	–	–
Digenea						
*Otodistomum* sp.	A	I	5.1	1.2 ± 0.4	–	–
Cestoda						
*Aporhynchus* sp.	A	SV	5.9	1.2 ± 0.4	–	–
*Plesiorhynchus* sp.	A	SV	17.8	5.6 ± 4.9	–	–
*Hepatoxylon* sp.	L	Me	5.1	1.3 ± 0.5	53.8	1.7 ± 1.4
Trypanorhyncha gen. sp.	L	Me	–	–	7.7	1.0 ± 0
Cestoda unidentified		SV	12.7	2.2 ± 2.1	–	–
Nematoda						
*Anisakis* sp.	L	I, M	13.3	1.0 ± 0	15.4	1.5 ± 0.5
*Mooleptus rabuka*	A	I	–	–	7.7	1.0 ± 0
Copepoda						
*Lernaeopodidae* gen sp.	A	B	2.5	1.0 ± 0	–	–
*Neoalbionella* sp.	A	F	12.7	1.1 ± 0.5	–	–
Thecostraca						
*Anelasma squalicola*	A	S, E, F, Mo	13.6	2.4 ± 0	–	–
Annelida						
*Piscicolidae* gen sp.	A	S	0.8	1.0 ± 0	–	–

A = adult, E = eyes, F = fins, G = Gills, I = intestine, L = Larval stage, Me = mesenteries, Mo = Mouth, SV = Spiral valve, S = Skin.

Two members of Copepoda were found: Lernaeopodidae gen sp. and *Neoalbionella* sp. The latter taxon showed the highest prevalence in *E. granulosus*. One annelid was found parasitizing *E. granulosus*; this leech was the least common taxon, with a single individual in the whole sample.

In both host species, Cestoda was well represented in terms of the number of taxa and individuals. In *E. granulosus*, three of four Cestoda were found in the adult stage, including *Plesiorhynchus* sp., which had the highest prevalence and mean intensity (Table 1), and one at the larval stage (*Hepatoxylon* sp.). The larval *Hepatoxylon* sp. was common for both sharks, showing the highest prevalence in *A. nasutus*. Of the three taxa of Nematoda, *E. granulosus* harboured one unidentified species of *Anisakis*, while *A. nasutus* harboured *Anisakis* sp. and *Mooleptus rabuka*. The digenean *Otodistomum* sp. was found only in *E. granulosus*, with low prevalence and abundance.

Individuals of the cosmopolitan mesoparasite *Anelasma squalicola* (Thecostraca) were found in both the dorsal and caudal fins, eyes and mouth of *E. granulosus*.

**Figure 2.**
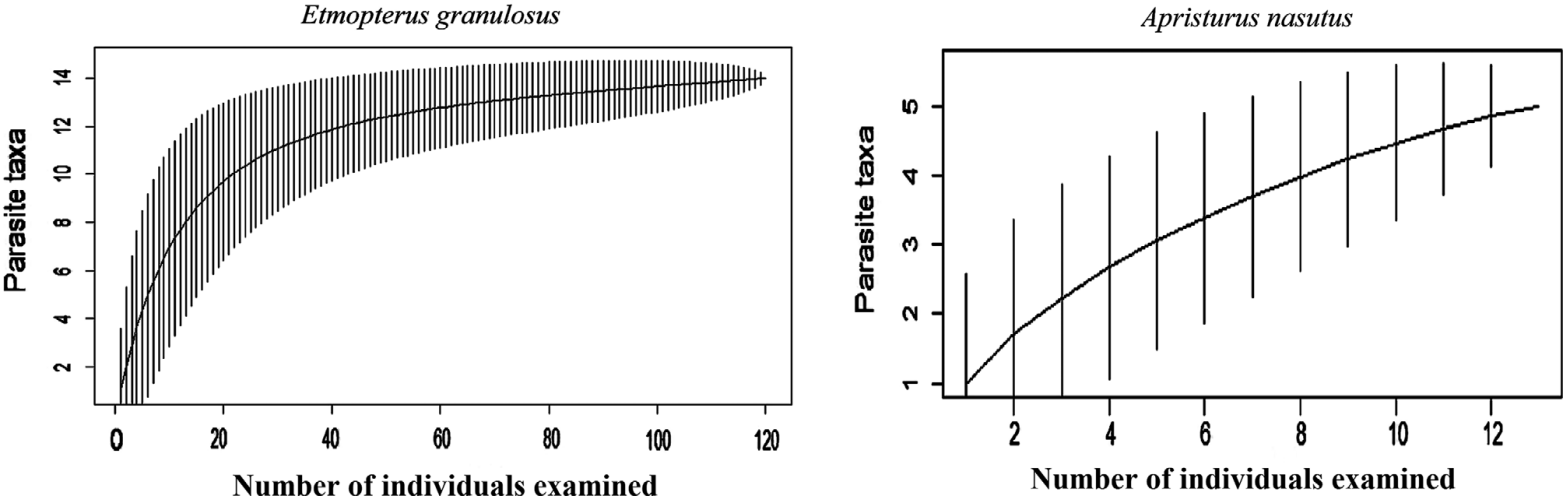
Species accumulation curves for both host species studied.

The sample size of *E. granulosus* allowed us to evaluate certain quantitative characteristics of the infection at the infracommunity and component levels (Table 2). Parasite species accumulative curves are shows in Figure 2, for both host species. The expected number of species ranged from 14.8 (Bootstrap) to 17.9 (Jackknife2) for *E. granulosus* and 5.9 (Bootstrap) and 6.9 (Jackknife2) for *A. nasutus* (Table 2).

**Table 2.** Quantitative characteristics of the metazoan parasites in two deep-sea sharks at component and infracommunity level.

	*E. granulosus*	*A. nasutus*
Component community		
Observed richness	14	5
Expected richness		
Chao	14.9 (*SE* = 2.3)	5.9 (*SE* = 1.7)
Jackknife 1	15.9 (*SE* = 1.4)	6.8 (*SE* = 1.3)
Jackknife 2	17.9	6.9
Bootstrap	14.8 (*SE* = 0.7)	5.9 (0.7)
Abundance	282	65
Diversity	1.9603 (*SD* = 0.064)	0.806 (*SD* = 0.121)
Evenness	0.738	0.501
Infracommunity		
Richness	1.06 (±1.27)	0.92 (±0.86)
Abundance	2.35 (*SD* = 3.71)	5 (*SD* = 9.30)
Diversity	0.26 (*SD* = 0.32)	0.13 (*SD* = 0.15)
Evenness	0.42 (*SD* = 0.46)	0.32 (*SD* = 0.43)

*SE* = standard error, *SD* = standard deviation.

The richness, abundance and diversity were positively and significantly correlated with host length (*r* = 0.408, *p* < 0.001, *df* = 118; *r* = 0.436, *p* < 0.001, *df* = 118; *r* = 0.41, *p* < 0.001, *df* = 65, respectively). The abundance of some taxa (*Asthenocotyle* sp. (*r* = 0.322, *p* < 0.001, *df* = 118), Monocotylidae gen. sp. (*r* = 0.228, *p* = 0.012, *df* = 118), *Plesiorhynchus* sp. (*r* = 0.286, *p* = 0.002, *df* = 118), Lernaeopodidae gen. sp. (*r* = 0.184, *p* = 0.044, *df* = 118), *Neoalbionella* sp. (*r* = 0.237, *p* = 0.009, *df* = 118), and *A. squalicola* (*r* = 0.293, *p* = 0.001, *df* = 118)) was positively and significantly correlated with host length. The prevalence of infection was significantly correlated with host length only for *Asthenocotyle* sp. (*r* = 0.94, *p* < 0.001, *df* = 3).

## Discussion

Hosts were obtained using the same fishing gear (deep-sea longline), on the same date and with the same fishing effort. Accordingly, the differences in the number of hosts obtained suggest differences in the relative abundance of each species. A similar result (higher abundance of *E. granulosus* than *A. nasutus*) was reported for the by-catch of the orange roughy, *Hoplostethus atlanticus*, in the SEPO [39].

This is the first study reporting the composition of the metazoan parasite community in the deep-sea sharks *E. granulosus* and *A. nasutus*. Most parasitological studies on deep-sea sharks have focused on the taxonomic description of a given parasite species or recorded the presence of parasite species in a given host, with few studies describing the species composition at the community level [13–15, 25, 28]. Previous studies regarding the parasites of *E. granulosus* and *A. nasutus* consist of taxonomic records.

For *E. granulosus*, there are six taxonomic records: *O. plunketi* from New Zealand [7, 22], *G. squali* from Chile, *A. tasmaniensis* and *P. etmopteri* from Australia [5, 11], *Neoalbionella* sp. from Juan Fernandez Archipelago, Chile [53], and *A. squalicola* from New Zealand [27, 60]. Accordingly, *Squalonchocotyle* aff. *spinacis, Asthenocotyle* sp., *Calicotyle* sp., *Monocotylidae* gen. sp., *Otodistomum* sp., *Aporhynchus* sp., *Plesiorhynchus* sp., *Hepatoxylon* sp., *Anisakis* sp., Lernaeopodidae gen sp. and *Piscicolidae* gen. sp. represent new records for *E. granulosus.* The *Neoalbionella* sp. now found could be the same taxon reported by Rodríguez et al. [53]. Unfortunately, males were not found, and they are necessary for the correct identification of the species; therefore, *Neoalbionella* sp. and *A. squalicola* represent new locality records. For *A. nasutus*, the only previous record is the nematode *M. rabuka* [54]; thus, the four remaining taxa (*Microbothrium* sp. *Hepatoxylon* sp., Trypanorhyncha gen. sp. and *Anisakis* sp.) represent new records for *A. nasutus.*

The most common taxon of parasites in elasmobranchs is Platyhelminthes, followed by Arthropoda, Nematoda, Annelida and Acanthocephala [9]. Our data align well with this pattern for both host species analysed. These authors also suggest that the richest group in the Platyhelminthes is Cestoda, followed by Monogenea and Digenea.

Parasitological studies are available for seven species of *Etmopterus*; of these studies, only three include quantitative data, but only for *E. spinax* [14, 25, 28]. Our data suggest that *E. granulosus* shows the highest richness among members of this genus (14 taxa) based on the published quantitative data (Supplementary material), but the integration of published taxonomic information (14 articles from seven localities) indicates the presence of 23 species, with Cestoda being the best represented group (14 taxa) (Supplementary material). With regard to Monogenea, *E. granulosus* showed the highest richness, with four taxa. Monogeneans have been reported only for two members of the *Etmopterus* genus. In regard to *A. nasutus*, the highest richness corresponds to Cestoda (two taxa) and Nematoda (two taxa). Among ectoparasites of elasmobranchs, Copepoda is the most diverse group, followed by Monogenea [9]. Our data show the opposite pattern: Monogenea was the richest group in *E. granulosus*. Copepoda was represented by two taxa, but specimens of *Neoalbionella* sp. seem in fact to be members of two species (Castro pers. comm.).

A different picture is evident for members of *Apristurus.* Taxonomic studies are available for seven species. The integration of taxonomic papers indicates richness ranging from one to two species rather than the five taxa found here. Any conclusion about richness in *A. nasutus* must be drawn with caution due to our small sample size and the absence of quantitative data for other members of the genus. However, the sample effort for both species captures the expected richness (Table 2).

Many factors have been proposed as drivers of the structure of parasitic communities, among others the diet of the host (generalist versus specialist predators), ontogenetic changes in the diet, prey availability (intermediate hosts) [50], as well as habitat, host behaviour (migratory versus sedentary, schooling versus non-schooling) and host density. Environmental factors, such as depth and water temperature, also influence community structure [19, 48, 50]. Recently [36], determinants of parasite species richness were evaluated, but no clear conclusions were obtained. Based on a meta-analysis, Kamiya et al. [26] suggest four potential universal predictors of richness, and three of them (host body length, geographical range size and population density) were adequate predictors, while latitude was not. Our results for *E. granulosus* are in partial accordance with those postulated, showing that a greater geographical range was related to higher parasitic richness. With respect to density, no data on host density are available, but because both species were obtained with the same fishing gear and sampling effort, this suggests that *E. granulosus* has a higher density than *A. nasutus*. However, when host length was evaluated, our results showed the opposite pattern to that expected according to the predictions of Kamiya et al. [26], as the mean length of *A. nasutus* was higher than that of *E. granulosus.* The positive and significant correlation found between host length and richness for *E. granulosus* suggests that for a given species, length is a suitable predictor of richness.

Six of the 14 taxa found in *E. granulosus* (*Asthenocotyle* sp., *Monocotylidae* gen. sp., Lernaeopodidae gen sp., *Neoalbionella* sp., *A. squalicola* and *Plesiorhynchus* sp.) showed a positive and significant correlation between abundance and host length (as a proxy for host age), suggesting cumulative infection with age or a colonisation rate that is higher than the mortality rate [47]. The prevalence of infection was significantly correlated with host length only for *Asthenocotyle* sp.

Despite the high diversity at the community component level for *E. granulosus*, at the infracommunity level, the richness and diversity showed low values that were similar to those reported for members of *Etmopterus* in other localities (Supplementary material).

The four taxa of monogenean recorded from *E. granulosus* represent unusually high richness for this group in deep-sea sharks. Just two species were recorded in 37 specimens of *E. spinax* [28], and Isber et al. [25] found only one species in a sample of 59 specimens. Moreover, Klimpel et al. [30] listed the parasite fauna of 30 shark species; of those, just three (*Hexanchus griseus*, *Etmopterus spinax* and *Dipturus oxyrinchus*) harbour two species of Monogenea, and the remaining 27 species harbour one or no species. A similar pattern was suggested [42, 43] in deep-sea teleosts in the SEPO. Although a small proportion of deep-sea teleosts and sharks have been studied, our results and those of Ñacari et al. [42] could be evidence that higher Monogenea richness in the SEPO is driven by environmental conditions.

Monogeneans found in *E. granulosus* belong to Hexabothriidae, Microbothriidae and Monocotylidae. Members of the Hexabothriidae and Monocotylidae are parasites of elasmobranchs and holocephalans [6, 12], whereas Microbothriidae are parasites of elasmobranchs only [57].

Two copepods were found in *E. granulosus*. Unfortunately, all the individuals found were females, and it was thus not possible to identify them to lower taxonomic levels. Both taxa belong to the Lernaeopodidae family. Members of the *Neoalbionella* genus are parasites of sharks of the Etmopteridae and Pentanchidae families [4, 24, 35, 53, 55].

The monotypic genus *Anelasma* is the only barnacle parasite of vertebrates [51]. This crustacean has been considered to be an ectoparasite [32], but it can be considered to be a mesoparasite, since it is partially embedded and partially exposed to the environment [9, 60]. *A. squalicola* (and also *Anelasma* sp.) has been reported only in sharks belonging to Etmopteridae [30], suggesting high host specificity.

Both presence of the adult and larval stages of endoparasites indicates that the studied sharks may function as final as well as intermediate hosts. Although the predators of *E. granulosus* and *A. nasutus* are unknown, small sharks are an important component of the diet of larger sharks; for example, small sharks such as *Galeus melastomus* and *Etmopterus spinax* are preyed upon by *Dalatias licha* [38].

Larval cestodes (*Hepatoxylon* sp. and Trypanorhyncha gen. sp.) can reach the adult stage in shark predators. Members of the *Hepatoxylon* genus (found in both hosts) are common parasites in teleosts (larval forms) and elasmobranchs (larval and adults) and have been reported from more than 40 species of sharks (see [49]). They have not been reported in sharks of the *Etmopterus* and *Apristurus* genera. The low prevalence and mean intensity in *E. granulosus* suggest accidental infection, but the high prevalence and mean intensity in *A. nasutus* suggests that this species is an intermediate host for this cestode. The lack of dietary data for these sharks prevents any conclusion about the life cycle of this worm. Merluccids and macrourids are part of the diet of *E. granulosus* in New Zealand [17]. In the SEPO, the south Pacific hake (*Merluccius gayi*), the Patagonian grenadier (*Macruronus magellanicus* (Merlucciidae)) and the bigeye grenadier *Macrourus holotrachys* (Macrouridae) are parasitized by larval *Hepatoxylon trichiuri* with high prevalence (>60% in *M. magellanicus*) [42, 45, 46], suggesting that *E. granulosus* may be infected with this larval cestode by preying on these fish species.

Although the life cycles of adult cestodes found in *E. granulosus* (*Aporhynchus* sp. and *Plesiorhynchus* sp) are not known, the feeding habits of *E. spinax*, suggest that the calanoid *Calanus finmarchicus* may be the first intermediate host for *Aporhynchus norvegicus*, while *Meganyctiphanes norvegica* is the second intermediate host [28].

*Aporhynchus* sp. and *Plesiorhynchus* sp. present some degree of host specificity. Currently, the trypanorhynch genus *Aporhynchus* comprises four species that infect three etmopterids: *A. norvegicus* and *A. menezesi* in *E. spinax, A. tasmaniensis* in *E. granulosus* (= *baxteri*) and *A. pickeringae* in *E. pusillus*; [40], but *Aporhynchus* sp. was detected in *Deania profundorum*, a member of Centrophoridae [10]. *Plesiorhynchus* includes three species; all of them have been found in deep-sea sharks, *P*. *campbelli* was found in *Apristurus* sp. [5] and in the etmopterids: *E. princeps* was infected with *P*. *brayi* and *E. lucifer,* and *E. granulosus* with *P. etmopteri* [5, 10].

Of the four nematodes found, two belong to the *Anisakis* genus. Adult *Anisakis* parasitize marine mammals, and larval anisakids are rare in sharks [28]. As stated by Henderson et al. [20], they must be considered accidental parasites; however, their presence has been reported in *Prionace glauca* [20], *Squalus acanthias* [21], *Centrophorus squamosus* [13], and *E*. *spinax* [28], as well as in the blackmouth catshark (*Galeus melastomus* (Pentanchidae)) [14, 15].

In *A. nasutus*, two nematodes were found: *Anisakis* sp. and *Mooleptus rabuka.* The latter species has been found in *Apristurus fedorovi* and *Apristurus japonicus* [2, 37]. Larval forms, presumed members of this nematode genus, have been found in the brain of the deep-water teleost *Cyclothone atraria* [33].

Despite its low prevalence and abundance, the presence of the digenean *Otodistomum* sp. cannot be the result of accidental infections; members of this genus have elasmobranchs as main hosts [9], and they have been reported in *E. granulosus* (and *E. baxteri*) [7, 22] and other members of *Etmopterus*, such as *E. princeps* [18] and *E. spinax* [25]. Their low abundance could be related to the low availability of intermediate hosts, such as some teleost fishes, the second intermediate host for this digenean [52].

## Conclusions

The present study analysed for the first time the composition of the metazoan parasite communities in two deep-sea sharks from the SEPO. The richness found in *E. granulosus* is the highest among members of the *Etmopteru*s genus. Twelve of the 14 parasite taxa found represent new records for this species, whereas for *A. nasutus*, four of five parasites represent new host records. The species composition for both host species analysed showed a pattern similar to that reported for other elasmobranchs, i.e., higher richness of Platyhelminthes, mainly Cestoda and also Monogenea. The diversity of Monogenea parasitizing *E. granulosus* is higher than that found in previous reports for elasmobranchs, and a pattern similar to that described for deep-sea teleosts in the SEPO is evident.

The presence of the cosmopolitan *A. squalicola* in *E. granulosus* suggests high connectivity in the deep sea, at least in the Southern Hemisphere.

## Supplementary Material

Tables The Supplementary Material is available at https://www.parasite-journal.org/10.1051/parasite/2018054/olm.
